# Enhancing the quality of lentil proteins via combination with whey proteins based on a dual process: a novel strategy through the incorporation of complexation and fermentation

**DOI:** 10.1007/s10068-024-01647-4

**Published:** 2024-07-04

**Authors:** Mohammad Alrosan, Thuan-Chew Tan, Azhar Mat Easa, Sana Gammoh, Muhammad H. Alu’datt, Stan Kubow, Ali Madi Almajwal, Ammar A. Razzak Mahmood, Ali Al-Qaisi, Hiba Bawadi

**Affiliations:** 1 Food Technology Division, School of Industrial Technology, Universiti Sains Malaysia, 11800 Gelugor, Penang Malaysia; 2 Applied Science Research Center, Applied Science Private University, Al-Arab St. 21, Amman, 11931 Jordan; 3 Renewable Biomass Transformation Cluster, School of Industrial Technology, Universiti Sains Malaysia, 11800 Gelugor, Penang Malaysia; 4 Department of Nutrition and Food Technology, Faculty of Agriculture, Jordan University of Science and Technology, P.O. Box 3030, Irbid, 22110 Jordan; 5 School of Human Nutrition, Macdonald Campus, McGill University, 21,111 Lakeshore Road, Ste-Anne-De-Bellevue QC, Montreal, H9X 3V9 Canada; 6 Department of Community Health Sciences, College of Applied Medical Sciences, King Saud University, P.O. Box 10219, 11433 Riyadh, Saudi Arabia; 7 Department of Agricultural Biotechnology, Faculty of Agricultural Sciences and Technology, Palestine Technical University-Kadoorie, Jaffa Street, P.O. Box 7, Tulkarm, Palestine; 8 Department of Pharmaceutical Chemistry, College of Pharmacy, University of Baghdad, Bab-Al-Mouadam, Baghdad, 10001 Iraq; 9 Department of Food Science and Nutrition, Faculty of Agriculture, Jerash University, Jerash, Jordan; 10 QU Health, College of Health Sciences, Qatar University, P.O. Box 2713, Doha, Qatar

**Keywords:** Lentil proteins, Whey proteins, Fermentation, Complexation, Water kefir grains

## Abstract

In recent years, there has been a growing interest in developing a distinguished alternative to human consumption of animal-based proteins. The application of lentil proteins in the food industry is typically limited due to their poor solubility and digestibility. An innovative method of balancing lentil-whey protein (LP-WP) complexes with higher-quality protein properties was established to address this issue, which coupled a pH-shifting approach with fermentation treatment. The results showed that microorganisms in the water kefir influenced the quality of protein structures and enhanced the nutritional values, including increasing the total phenolic compounds and improving the flavor of fermented LP-WP complexes. The protein digestibility, pH values, microbial growth, total soluble solids, and total saponin and phenolic contents were hydrolyzed for 5 days at 25 °C. The FTIR spectrophotometer scans indicated significant (*P* < 0.05) changes to the secondary protein structure components (random coil and α-helix). This study showed that combining pH-shifting with fermentation treatment improves lentil and whey proteins’ structure, protein quality, and nutritional benefits.

## Introduction

Plant proteins are increasingly used in food items instead of animal proteins due to their greater long-term viability and less negative environmental impact. Lentils are excellent plant-based protein sources. In contrast to other plant-based foods, lentils have a comparatively high protein content, and they are considered a good choice because of their excellent nutritional value, high lysine content, balanced amino acid profile, and low price (Alrosan et al., 2022). In addition, lentil proteins have reportedly shown some biological activity that might positively affect a person’s health, such as gut microflora modulation, blood pressure lowering, and antioxidant activity. However, the general acceptance of lentil proteins in food applications remains restricted, primarily due to their weak solubility in water (Alrosan et al., 2024). Notably, they include considerable quantities of globulin, and the procedures for separating them include denaturation and aggregation of proteins, acid precipitation, and alkaline extraction (Miranda et al., 2023). The inability of lentil proteins to dissolve in water negatively impacts their digestion (Alrosan et al., 2024). For example, the solubility of the chickpea protein increased, as did the digestibility, after 16 h of fermentation by lactic acid bacteria (LAB) (Liu et al., 2023).

Numerous studies have reported that the modification of protein–protein interactions through pH shifting is an essential variable in enhancing protein solubilities, such as rice protein-casein protein (Wang et al., 2018), lentil protein-whey protein (Alrosan et al., 2021), and lentil protein-casein protein (Alrosan et al., 2023). The water solubility of proteins substantially affects their functionality and nutritional properties. Water solubility affects the ability of proteins to interact with other molecules and perform their biological functions. The hydrophobic or hydrophilic nature of amino acid residues within a protein can determine its water solubility. However, according to a study by Lui et al. (2023), the fermentation process involving LAB may impact the protein structure of chickpea protein. This impact increased solubility and digestibility throughout the fermentation (Al‐Qaisi et al., 2024).

Furthermore, water kefir fermentation could also contribute beneficial compounds, such as vitamins and amino acids. These compounds can contribute to improved nutritional value and health benefits of the fermented product. On the other hand, it was stated by Jiang et al. (2022) and Alrosan et al. (2023) that the utilization of dual processes such as pH-shifting and ultrasonic processes (Jiang et al., 2022) and complexation-based pH-shifting and fermentation (Alrosan et al., 2023), This technique has a direct effect on proteins’ functional and structural properties. For example, changes in the pH level can alter the shape of a protein, affecting its ability to bind to other molecules or carry out its specific function. Additionally, extreme temperature changes can cause proteins to denature and lose functionality (Alrosan et al., 2022). These dual processes have improved protein solubility, enhanced emulsifying properties, and increased digestibility (Alrosan et al., 2024). These findings suggest that using dual processes can be a promising approach for improving the functional properties of proteins in various food applications. This innovative strategy can potentially improve the nutritional value of lentil proteins, often limited by their low levels of essential amino acids and digestibility. Combining it with whey proteins, which are rich in these amino acids, could offer consumers a more complete source of protein. Additionally, complexation and fermentation technologies could help increase the bioavailability and digestibility of the proteins, further enhancing their nutritional benefits.

This study investigated the relationship between modifications in structural and functional properties and differences in composition and multilevel structure of protein complexes based on lentil and whey-produced protein–protein interaction following the fermentation of water kefir grains for 5 days. Water kefir has been recognized as an essential source of fermenting microorganisms, particularly yeasts, LAB, and acetic acid bacteria (AAB). They are commonly found in fermented foods such as yogurt, kefir, and sauerkraut. Additionally, LAB and AAB have been shown to have potential health benefits, such as improving digestion and boosting the immune system. The protein structure of the lentil-whey protein (LP-WP) complexes was analyzed using various techniques. The secondary protein structure components, protein conformation, and tertiary protein structure were investigated using FTIR and UV–vis spectroscopy. Besides, the microbial growth curve, pH values, total phenolic compounds, and digestibility of LP-WP complexes were determined to show the fermented complex protein has the potential to be a high-quality source of complex protein.

## Methods and materials

Kefir and lentil seeds (*Lins culinaris*) were purchased from the iHerb online store (Moreno Valley, California, USA). Whey protein isolates were purchased from a store in Penang (GNC, Penang, Malaysia). This storage condition prevented any potential spoilage or degradation of the materials. Additionally, the refrigeration unit provided a stable environment for storing the materials.

### Preparation of lentil proteins

Lentil protein with protein content (62.5%) was isolated based on the procedure mentioned by Alrosan et al. (2021). The lentil seeds were subjected to 2 rounds of washing with cold water and then placed at 25 °C for 24 h to decrease the saponin (Alrosan et al., 2023). The washed lentil seeds were ground into fine powders with a particle size smaller than 0.5 mm using a rotating mill (Retsch, ZM 300, Haan, Germany). The flour obtained was gathered in airtight bags and kept at a temperature of 4 °C until the protein extraction process through consecutive drying. The fine lentil flour was dissolved in distilled water at 10:1 (w/v). A digital magnetic stirrer (JoanLab, SH-4, USA) was used to stir the suspension, and the pH level was modified to pH 9.5 by adding NaOH (0.1 M). The alkaline extraction process allowed for the separation of lentil proteins from other components present in lentil grains, and it helped solubilize the proteins. The suspension was stirred (1,000 rpm) for 2 h at 40 °C. The pH was checked every 30 min during this step. Afterward, the suspension was centrifugated (Kubota, S700TR, Tokyo, Japan) at 8500×*g* for 15 min at 23 °C. The liquid portion was collected, adjusted to a pH of 4.5 using hydrochloric acid (0.1 M), and left undisturbed for a whole night before being subjected to centrifugal (1590×*g*) for 30 min. The solid was collected and freeze-dried (Büchi, R-220, Flawil, Switzerland). The lyophilized material was processed, transferred into plastic bags, hermetically sealed, and kept at a temperature of 4 °C until it was combined with lentil protein.

### Preparation of water kefir

The current investigation involved preparing a batch of water kefir using the method described by Alrosan et al. (2023). A kefir aqueous solution was produced through a combination of kefir grains (5%), brown sugar (10%), and distilled water. The solution was then incubated at room temperature (25 °C) for 72 h. The resulting mixture was then filtered, and the kefir grains were removed. The resulting liquid was ready to water kefir fermentation.

### Preparation of fermented protein complex

The pH-shifting procedure is a commonly used method based on Alrosan et al. (2021) for obtaining complex protein solutions. The LPs and WPs (1%, w/v) were mixed in distilled water to create the initial protein solution. The pH of the mixture was then adjusted to a highly alkaline level (pH 12) using a 0.5 M NaOH solution. After an hour, the pH was readjusted to neutral using a 0.1 M HCl solution, forming the protein complex. This procedure allows for the purification and stabilization of different types of proteins. In a flask with a capacity of 250 mL, a solution consisting of complex protein (1% w/v), water kefir (5 mL), and distilled water (95 mL) was combined to carry out the fermentation process. The solution was incubated in a refrigerated incubator at 25 °C for 5 days. During the incubation period, the solution was shaken three times a day to maintain the viability of the fermenting bacteria. The samples were obtained and assessed every day.

### Determination of pH and total soluble solids (TSS)

A pH meter was calibrated using standard buffer solutions before each measurement to ensure accuracy. The results were recorded and used to determine the acidity of the fermenting medium containing the LP-WP complex. In addition, using a digital refractometer, the fermenting medium’s TSS (expressed as °Brix) was also determined (Alrosan et al., 2021).

### Determination of protein digestibility

The digestibility of protein samples was determined according to the method mentioned by Almeida et al. (2015) with slight modifications. The protein mixture was prepared by combining 250 mg of the protein sample with 15 mL of 0.1 M HCl solution containing 1.5 mg/mL of pepsin. The mixture was then incubated at 37 °C for 3 h with constant shaking. After the incubation period, the reaction was stopped by adding 0.5 M NaOH to raise the pH to 7.0. The resulting solution was then added to 10 mL of solution containing pancreatin (10 mg), 0.005 M sodium azide (1 mL), and 0.2 M phosphate buffer (pH 8.0). The solution was then incubated at 37 °C for 24 h with constant shaking. Finally, the resulting mixture was centrifuged, and the supernatant was collected for further analysis. The nitrogen content of the sample was calculated by subtracting the nitrogen content of the blank sample and supernatant and dividing the total nitrogen content of the sample. This method was based on the Kjeldahl method (Method 930.29) (AOAC, 2012).

1 $${\text{Protein digestibility}}\;\left( \% \right) = \left[ {\left( {{\text{N}}_{{\text{S}}} - {\text{N}}_{{\text{B}}} } \right)/{\text{N}}_{{\text{T}}} } \right] \times {1}00\%$$

where, N_T_ is the total nitrogen content of the sample, and N_B_ and N_S_ are the nitrogen content of the blank sample and supernatant, respectively.

### Determination of secondary protein structure

The secondary protein structure of the protein sample was conducted based on the procedure described by Alrosan et al. (2021) using an FTIR spectrometer (Shimadzu, IRAffinity-1S, Kyoto, Japan). Protein samples were dried before analysis, and grinding was obtained to obtain homogeneous protein powders of less than 20 mm. This step is critical to ensure uniformity and facilitate accurate spectrum scanning. The homogeneous protein powders (60 mg) were placed on the diamond at the center of the spectrometer for scanning. The spectral range was measured between 400 and 4000 cm^−1^. This range covers the infrared region and allows for detecting characteristic vibrations and absorption bands of the protein samples. At the same time, the scanning interval was set to 4 cm^−1^. The normalized data of the amide I region, specifically the region spanning 1600–1700 cm^−1^, was subjected to baseline correction. Baseline correction helps to remove any systematic or instrumental variations in the spectra that may interfere with the analysis. The amide I region is essential in the FTIR spectra of proteins and contains information about the protein’s secondary structure. The components of secondary protein were calculated based on the amide I band. Specifically, the following components were analyzed and expressed as percentages of the amide I band: β-sheet (1600–1639 cm^−1^), random coil (RC, 1640–1649 cm^−1^), α-helix (1650–1660 cm^−1^), and β-turn (1661–1699 cm^−1^) (Alrosan et al., 2023).

### Determination of glucose, sucrose, and fructose

The glucose, sucrose, and fructose levels were determined using HPLC (Agilent, 1200 series, New Jersey, USA). The sugars were quantified by mixing the protein samples (1 mL) with distilled water (1 mL), followed by vertexing for 10 min. The mixtures were centrifuged at 15,000 rpm for 10 min, and the supernatants were collected. The supernatants’ glucose, sucrose, and fructose contents were separated using a Cosmosil Sugar-D column (4.6 × 250.0 mm). The mobile phase comprised water and acetonitrile (75:25, v/v) with an injection volume of 20 µL and a 1.2 mL/min flow rate. A refractive index detector detected the sugars. The standard curves were constructed using absorbance values and plotted against each sugar’s known concentrations. The resulting standard curves determined the fermented samples’ glucose, sucrose, and fructose concentrations.

### Determination of total phenolic compounds

The solutions’ total phenolic content was measured per the methodology described by Alrosan et al. (2023). The standard curve was established by plotting the absorbance values against the concentrations of gallic acid 10 mg/100 mL to determine the total phenolic content in various samples by comparing their absorbance values to the standard curve. The sample preparation involved mixing 100 µL of the protein samples with 500 µL of Folin-Ciocalteu reagent and 8.4 mL of distilled water in a 50 mL glass test tube. The mixtures were rested for 4 min before adding 1 mL of sodium carbonate solution (5%). The resulting solutions were mixed thoroughly and left to react for 60 min at room temperature. After the reaction, the absorbance of the solutions was recorded at 725 nm using a spectrophotometer (Shimadzu, UV-3600, Kyoto, Japan). The concentration of total phenolic compounds in the protein samples was determined using a standard curve generated with gallic acid as the standard.

### Determination of phenolic compounds

The method described by Alrosan et al. (2023) was used to extract the phenolic compounds from the protein samples (1 mL) using methanol (8 mL) assisted by ultrasonics treatment at 35 °C. After 3 min of sonication, the mixtures were kept at 4 °C in a refrigerator until the residues turned white. The mixtures were centrifuged at 10,000×*g* for 15 min to separate the supernatants. The collected supernatants were stored in HPLC vials while waiting for HPLC sample injection.

The mobile phases used in the HPLC analysis were acetonitrile (mobile phase A) and 1% (v/v) acetic acid solution (mobile phase B). The gradient profile was as follows: at the start of the analysis, the mobile phase consisted of 95% A and 5% B. After 25 min, the proportion of A decreased to 85%, while B increased to 15%. The composition was then changed to 78% A and 22% B at the 42nd min and 64% A and 36% B at the 60th min. At the 65th min, the mobile phase composition was reverted to 95% A and 5% B. This gradient profile helps optimize the separation of phenolic compounds with plus C18 (4.6 × 250.0 mm) with an injection volume of 40 µL, and the flow rate was set at 0.7 mL/min. The samples’ detection wavelengths were read at 254 and 272 nm at the specific retention time for each phenolic compound.

Working standards are prepared by diluting the stock solution of the phenolic compounds to appropriate concentrations. These working standards are then used to construct standard curves. Standard curves are created by analyzing different concentrations of these standards and plotting the corresponding peak areas. The resulting curve is used to quantify the concentration of phenolic compounds in samples by comparing their peak areas or heights to the standard curve.

### Determination of total saponins content (TSC)

TSC was determined for the protein samples based on the procedure described by Xiao et al. (2014) with some modifications. Glass tubes (50 mL) were utilized to combine protein samples (400 µL), vanillin-glacial acetic acid (5%, 200 µL), and 800 µL of perchloric acid. The mixtures were then heated in a water bath at 70 °C for 15 min. After cooling, glacial acetic acid (500 µL) was added to the mixes and vortexed for 30 s before measuring the absorbance of the solutions at 546 nm using the UV-3600-spectrophotometer. A standard curve was prepared using oleanolic acid at concentrations ranging from 6.25 to 600 µg/mL. TSC was expressed as milligrams of oleanolic acid equivalent per 100 g protein complex (mg OAE/100 g protein complex).

### Statistical analysis

Statistical analyses were performed using SPSS version 23.0 (IBM, Chicago, USA). Duncan’s multiple range test was used for pairwise comparisons of means. ANOVA was used to analyze differences between the means of three or more groups. A significance level (*P* < 0.05) was used, meaning the differences were considered statistically significant if the statistical test’s *P*-value was less than 0.05.

## Results and discussion

### Effect of water kefir fermentation on the pH and TSS of LP-WP fermenting medium

The pH of the fermenting medium decreased throughout the fermentation period (Table 1) due to the fermenting microorganisms in the water kefir produced. After 24 h of fermentation, the LP-WP fermenting medium’s pH decreased from 6.73 to 4.05. The pH of the LP-WP fermenting medium was observed to reach 3.60 during the final fermentation phase. This decrease in pH could be attributed to the production of acidic by-products by the fermenting microorganisms during fermentation.

**Table 1:** Changes in the pH, total soluble solids (TSS, °Brix), protein digestibility (%), total saponins content (TSC, mg OAE/100 g protein complex), and sugar profile (g/L) of unfermented LP-WP complex (Day 0) and water kefir-fermented LP-WP complexes (Day 1 to 5)

Parameters	Fermentation period (Day)						*P* value
	0	1	2	3	4	5	
pH	6.73 ± 0.02^a^	4.53 ± 0.02^b^	4.05 ± 0.02^c^	3.82 ± 0.02^d^	3.72 ± 0.02^e^	3.60 ± 0.01^f^	*P* < 0.05
TSS	1.76 ± 0.05^a^	1.40 ± 0.00^b^	1.30 ± 0.00^c^	1.26 ± 0.05^cd^	1.20 ± 0.00^de^	1.16 ± 0.05^e^	*P* < 0.05
Protein digestibility	81.69 ± 1.74^c^	84.44 ± 1.26^c^	88.84 ± 1.90^b^	90.41 ± 0.13^ab^	92.17 ± 0.72^ab^	93.00 ± 0.53^a^	*P* < 0.05
TSC	25.60 ± 0.57^a^	24.86 ± 0.68^b^	22.10 ± 0.40^c^	20.96 ± 0.51^d^	19.01 ± 0.52^e^	18.10 ± 0.72^e^	*P* < 0.05
Sugars							
Fructose	0.54 ± 0.01^c^	2.28 ± 0.06^a^	1.03 ± 0.03^b^	0.47 ± 0.02^d^	0.19 ± 0.01^e^	0.06 ± 0.00^f^	*P* < 0.05
Glucose	0.07 ± 0.01^f^	1.28 ± 0.04^b^	2.82 ± 0.10^a^	1.08 ± 0.04^c^	0.49 ± 0.02^d^	0.19 ± 0.01^e^	*P* < 0.05
Sucrose	4.45 ± 0.19^a^	0.87 ± 0.03^b^	0.12 ± 0.01^c^	0.05 ± 0.01^c^	ND	ND	*P* < 0.05

The data presented in the table represent the mean ± standard deviation (n = 3). Values with different superscripts within the same row are statistically significant from each other (*P* < 0.05). ND represents not detected. OAE represents the oleanolic acid equivalent

**Fig. 1:**
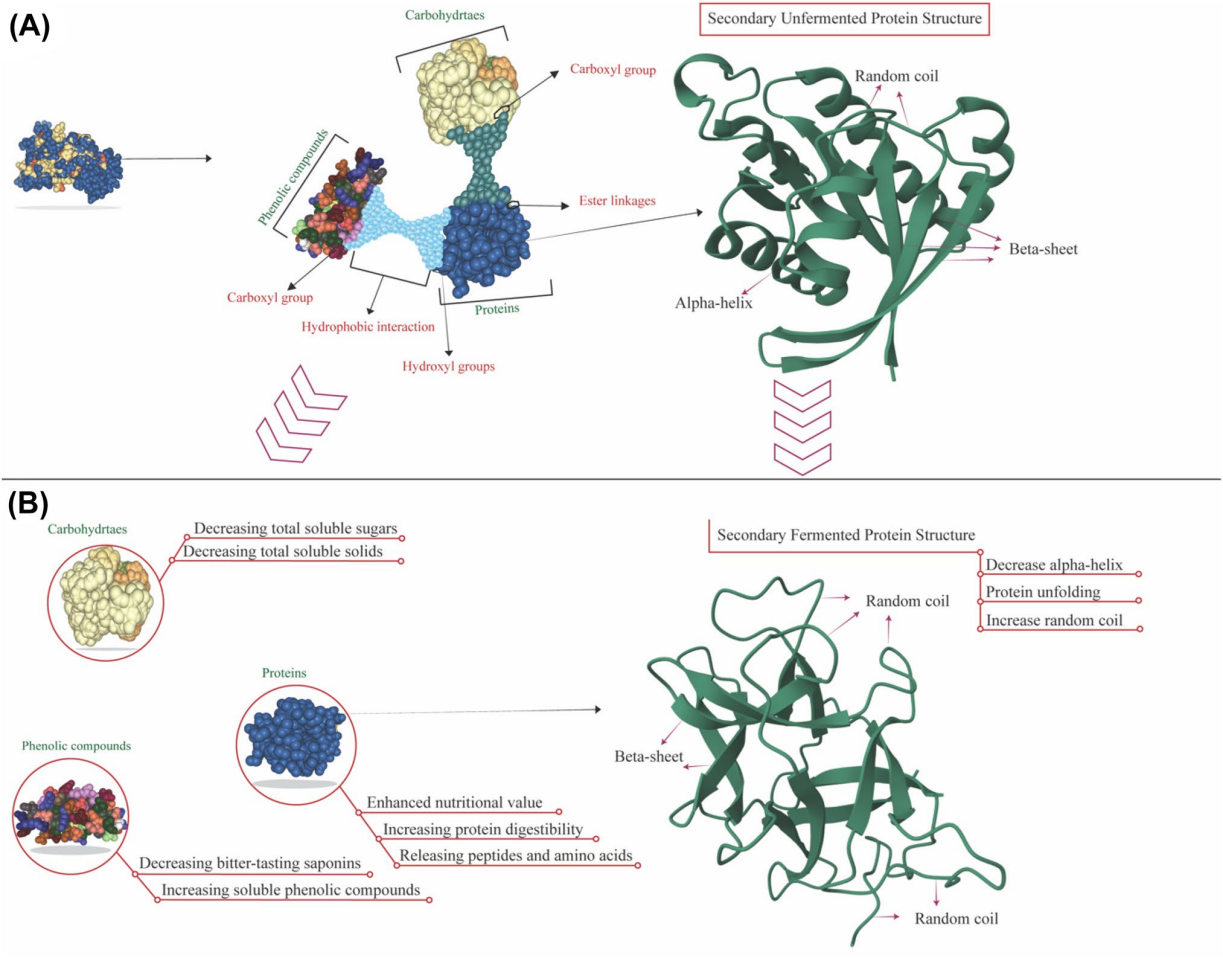
A schematic diagram illustrating the changes to the complex proteins during the fermentation of water kefir **A** illustrates the complex proteins based on the whey and lentil proteins and the associations between the carbohydrates, proteins, and phenolic compounds. **B** Illustrated the fermented complex protein by water kefir due to the breakdown of the relationships between the carbohydrates, proteins, and phenolic compounds. This breakdown of relationships is crucial to understanding the nutritional composition and potential health benefits of water kefir

Protein degradation involves breaking complex proteins into their constituent amino acids (Fig. 1A). This process can be catalyzed by enzymes called proteases, produced by active fermenting microorganisms. As proteins are broken down, amino acids and NH_4_^+^ are released into the fermentation medium (Tepari et al., 2020). Moreover, amino acids can undergo decarboxylation reactions, producing organic acids as by-products, further acidifying the medium. During lactic acid fermentation, LAB utilize sugars as energy sources and produce lactic acid as a metabolic by-product. The accumulation of lactic acid in the fermentation medium contributes to reducing pH. Lactic acid is a weak organic acid that dissociates to release hydrogen ions (H^+^), which acidifies the environment (Çabuk et al., 2018; Jia et al., 2021). Similar observations were reported in past studies (Alrosan et al., 2023; Jia et al., 2021), indicating the presence of *Lactobacillus* spp. contribute to the decrease in the protein pH following water kefir fermentation.

The TSS of the WP-LP fermenting medium was decreased significantly (*P* < 0.05) (Table 1) during the water kefir fermentation. The TSS of the fermenting medium decreased from 1.76 (initial value) to 1.26°Brix (Day 3). The decrease in TSS during water kefir fermentation can be attributed to several factors. One possible reason for the decline in TSS is the consumption of sugars by fermenting microorganisms in the water kefir. During fermentation, these microorganisms, which typically include a combination of bacteria and yeasts, metabolize the sugars present in the medium as a source of energy. As a result, the concentration of sugars in the solution decreases, leading to a decrease in TSS. At the end of the fermentation stage, TSS reached around 1.16°Brix.

These results refer to the fermenting microbial activity, mainly LAB (*Lactobacillus* spp.), AAB, and yeasts in water kefir. It was reported by Alrosan et al. (2023) that fermenting microorganisms could contribute to reducing the TSS from 1.5 to 1.13°Brix throughout the 5 days of water kefir fermentation. This reduction in TSS is attributed to these fermenting microorganisms’ conversion of sugars into organic acids. The study also found that the fermentation process increased the antioxidant activity of the final stage of fermentation. Besides, dos Santos et al. (2019) reported that the TSS of soymilk during 5 days of fermentation (25 °C) with kefir 4% (w/v) decreased from 1.88 to 1.45°Brix. The reduction of TSS may be attributed to the *Lactobacillus* and *Lactococcus* species (Alrosan et al., 2022; dos Santos et al., 2019).

### Effect of water kefir fermentation on protein quality of fermented LP-WP complexes

Protein digestibility refers to how proteins are broken down and absorbed in the digestive system. It is influenced by several factors, including the composition of the protein, its structural characteristics, and the presence of other substances that may hinder or facilitate digestion (Alrosan et al., 2022). The observed variation in digestibility in LP-WP complexes could be attributed to multiple factors. One factor could be the specific composition and structure of the LP-WP complexes themselves. Different proteins have varying susceptibilities to enzymatic breakdown, and complex formation between proteins can affect their accessibility to digestive enzymes (Fig. 1B).

The processing and fermentation conditions employed during the preparation of LP-WP complexes may also influence their digestibility. For example, specific processing techniques or product fermentation could alter the protein complexes’ structure or composition, affecting their digestibility. The digestibility of LP-WP complexes with protein digestibility values ranges from approximately 81.69 to 93.00% (Table 1). The protein digestibility of LP-WP complexes increased and reached 90.41% on the 3rd day of water kefir fermentation and then further increased to 93.00% on the 5th day. It was recently reported by Liu et al. (2023) that *Lactobacillus* fermentation has improved the digestibility of chickpea protein from 65 to 71% after 16 h of fermentation. Additionally, the fermentation by *Streptococcus bulgaricus* and *Lactobacillus* spp. can increase the protein digestibility of the chickpea flour from 70.5 to over 77.2%.

It can be expected that the protein digestibility of LP-WP complexes could increase during fermentation with water kefir. This expectation was based on the reported presence of fermenting microorganisms, particularly yeasts and LAB. Both can contribute to the enzymatic breakdown and digestion of proteins. Yeasts, such as *Saccharomyces*, can produce various proteolytic enzymes that hydrolyze proteins into smaller peptides and amino acids, thus enhancing protein digestibility (Fig. 1A). In addition, LAB can also produce proteases that aid in protein degradation (Rodrigues et al., 2016). A recent study by Alrosan et al. (2023) revealed that the digestibility of protein complex containing casein and lentil proteins has been reported to increase from 79.53 to 86.79% because the fermentation medium is rich in fermenting microorganisms. This increase in digestibility can be attributed to the breakdown of complex protein structures into simpler forms that are easier for the body to absorb. These findings suggested incorporating fermented foods into the diet may benefit protein digestion and absorption.

Insignificant (*P* > 0.05) changes in the digestibility of fermented LP-WP complexes were observed starting from the 3rd day until the end of the water kefir fermentation (Table 1). This result could be attributed to decreased protein cross-linking stimulated by antioxidants, phenolic contents, and saponins of non-nutritive compounds that can cause proteolytic attacks. The increase in digestibility of fermented protein complexes is associated with the hydrolysis of protein and non-nutritive compounds, such as phenolics, saponins, and anti-nutrients forming with protein complexes. Studies conducted by Pranoto et al. (2013) and Mugula et al. (2003) proved that *L. plantarum* has proteolytic activity.

Several studies by Alrosan et al. (2023) and Çabuk et al. (2018) have shown that phenolic compounds can interact with proteins and affect their digestibility. These interactions can lead to reduced enzymatic activity and hinder the accessibility of protein molecules to digestive enzymes. Phenolic compounds can form complexes with proteins, resulting in protein–phenolic interactions that interfere with the enzymatic breakdown of proteins during digestion.

During fermentation, microorganisms produce various enzymes, such as proteases, that catalyze the hydrolysis of proteins. These proteases cleave the peptide bonds between amino acids, releasing smaller peptide fragments and individual amino acids (Alrosan et al., 2022). The presence of amino acids and peptides can facilitate protein digestion because of increased solubility (Alrosan et al., 2022), improved enzyme specificity (Yang et al., 2020; Klaenhammer et al., 2005), and enhanced absorption (Jia et al., 2021). In this study, increased nitrogen content increased protein detestability during fermentation. Jia et al. (2021) carried out a study that demonstrated that fermentation led to a substantial rise in the degradation of ovalbumin and ovomucoid, attributed to the breakdown of peptide bonds (Fig. 1B). In addition, that study agreed with recent studies, whereby protein digestibility increases during fermentation using *Lactobacillus* spp. (Ayala-Niño et al., 2019). As previously reported by Çabuk et al. (2018), it was reported that the digestibility of pea protein concentrate increased after fermentation with *Lactobacillus plantarum*. Overall, fermentation can inhibit protein cross-linking, a process in which proteins form cross-links, resulting in a more complex and rigid protein structure. Cross-linked proteins are generally more resistant to enzymatic breakdown and can be less digestible. Fermentation processes, particularly those involving LAB, can produce enzymes that can break down these protein cross-links, leading to increased protein digestibility.

Overall, the fermentation of legumes can improve protein digestibility by reducing the levels of non-nutrient compounds, inhibiting protein cross-linking, and generating enzymes that facilitate protein breakdown. This transformation through fermentation enhances the nutritional value of legumes as a protein source. Studies by Çabuk et al. (2018), Chandra-Hioe et al. (2016), and Alrosan et al. (2022) reported that partial degradation and subsequent release of specific proteins can be attributed to bacterial proteases. The formation of saponin–protein complexes reduces the digestibility of proteins by saponins (Ahuja et al., 2015; Segal et al., 1970).

### Effect of water kefir fermentation on sugars content of fermented LP-WP complex

Brown sugars were responsible for the functional activity of water kefir (Alrosan et al., 2022), which is primarily composed of sucrose (more than 85%) and traces of glucose and fructose (dos Santos et al., 2018). The study observed a decline in sucrose content within the LP-WP complexes as fermentation time was extended. The sucrose content in the LP-WP complexes solution reduced to 0.11 ± 0.002, 0.12 ± 0.005, and 0.14 ± 0.005 g/L on the 2nd day of the fermentation, while glucose of the LP-WP complexes increased to 2.82 ± 0.10 g/L. Concurrently, the fructose of LP-WP complexes increases to 1.03 ± 0.0 g/L (Table 1).

It is reported that the sugars and fermentation conditions may influence the growth and colony formation of the LAB (Alrosan et al., 2023; Pranoto et al., 2013). In addition, different strains of LAB may exhibit variations in their sugar utilization and colony-forming abilities. The fermenting microorganisms reduced the total sugars determined during fermentation in the water kefir at the end of the fermentation. Sucrose, glucose, and fructose are the main components of the brown sugars found in the fermenting medium in this study. The microbial consortium, which typically includes a combination of bacteria (LAB and AAB) and yeast, possesses enzymes that can break down these sugars into simpler forms and utilize them as a carbon source for their growth and metabolic activities (Lynch et al., 2021). These results are consistent with those observed by Tu et al. (2019) and Martinez-Torres et al. (2017), which showed that fructose and glucose levels in the solution increased at the expense of sucrose throughout the 48 h of fermentation.

Moreover, Silva et al. (2009) used kefir seed to ferment different sugar sources, including molasses and brown sugar, to demonstrate antimicrobial activity during kefir fermentation. Brown sugar had the most potent antibacterial activity toward *Escherichia coli*, *Staphylococcus aureus*, *Shigella sonnei*, *Salmonella typhi*, and *Candida albicans*. During fermentation by yeasts and bacteria and their enzymes, monosaccharides, disaccharides, oligosaccharides, refined oligosaccharides, fructans, and lactose can be partially and fully degraded (Gänzle, 2020). LAB can hydrolyze raffinose-family oligosaccharides through sucrose-phosphorylase, α-galactosidases, and levansucrase activities (Pelletier et al., 2001).

### Effect of water kefir fermentation on protein structures of fermented LP-WP complex

The current study utilized FTIR spectroscopy to investigate the secondary protein structure of fermented LP-WP complexes within the amide I region over 5 days. Table 2 shows the percentage of the secondary structures identified in LP-WP complexes. The components of the unfermented LP-WP complexes were determined by α-helices (7.64%), β-turns (44.91%), RC (13.08%), and β-sheets (34.35%). The percentage of α-helix and RC exhibited alterations due to the duration of fermentation. These modifications influence the degradation of proteins and can lead to changes in their secondary (Fig. 1A), tertiary, and quaternary structures. The unfolding and rearrangement of protein chains can occur during fermentation, altering the overall conformation and organization of the degraded proteins. In contrast, the percentages of β-turns and β-sheets are insignificant (*P* > 0.05) during the fermentation by water kefir. It was reported by Carbonaro et al. (2012) that an increase in the percentage of β-sheet and protein digestibility can have an adverse effect.

**Table 2:** Changes in the percentage of secondary protein components (β-sheet, random coil (RC), α-helix, and β-turn) of unfermented LP-WP complex (Day 0) and water kefir-fermented LP-WP complexes (Day 1 to 5) based on FTIR measurements

Secondary protein components	Peak (cm^−1^)	Fermentation period (Day)						*P*-value
		0	1	2	3	4	5	
β-sheet								
	1,614.42	13.66	13.99	14.38	13.79	11.56	11.02	
	1,622.13	12.36	12.44	12.77	12.57	12.25	11.09	
	1,633.71	8.34	8.55	8.82	8.82	8.55	8.05	
β-sheet (Ʃ)		34.35^d^	34.97^c^	36.07^a^	35.17^b^	32.36^e^	30.16^f^	*P* > 0.05
RC (Ʃ)	1,645.28	13.08^f^	13.55^e^	14.47^d^	16.51^c^	18.34^b^	20.28^a^	*P* < 0.05
α-helix (Ʃ)	1,654.07	7.64^a^	6.29^a^	0.00	0.00	0.00	0.00	*P* < 0.05
β-turn								
	1,668.43	11.75	11.97	14.01	14.01	14.08	14.25	
	1,681.93	12.42	12.46	11.24	11.24	11.92	11.91	
	1,693.50	20.74	20.75	23.06	23.06	23.29	23.39	
β-turn (Ʃ)		44.91^e^	45.17^d^	49.44^a^	48.31^c^	49.28^b^	49.54^a^	*P* > 0.05
Ratio^A^		22.25	17.99	0.00	0.00	0.00	0.00	

The data presented in the table represent the mean ± standard deviation (n = 3). Values with different superscripts within the same row are statistically significant from each other (*P* < 0.05). LP-WP complex represents LP:WP at a ratio of 1:1.2

^A^Ratio of α-helix:β-sheet

There is no significant difference in the α-helices of the LP-WP complex during the first 2 days of the fermentation process using water kefir. However, on Day 3, the water kefir fermentation has a significant impact on the α-helices of the LP-WP complex. This impact is likely due to changes in the pH and nutrient availability in the fermentation medium, which can affect the protein structure. These findings suggest that the 3rd day of the water kefir fermentation is a critical stage in the fermentation process for understanding the structural changes of the LP-WP complex. Moreover, strong proof indicates that the deterioration of LP-WP complexes led to a significant (*P* < 0.05) increase in RC from 13.08 to 13.55% on the 1st day of fermentation and subsequently increased to 20.28% on the last fermentation. This upward course suggests that the LPs had consolidated.

Studies have shown that proteins with higher α-helix content tend to have lower digestibility rates than proteins with lower α-helix content. This hypothesis is because α-helix structures can restrict the accessibility of digestive enzymes to the peptide bonds, hindering the breakdown process (Alrosan et al., 2023; Wang et al., 2014). This reduction in ɑ-helix percentage is often accompanied by increased random coil or β-sheet components, which are more easily broken down by digestive enzymes. Additionally, the increased digestibility of proteins can lead to improved nutrient absorption and utilization by the body (Liu et al., 2023). Our findings agree with Yasar et al. (2020), Liu et al. (2023), and Alrosan et al. (2023). In contrast, industrial food processes involving extreme heating and pressure treatments, such as cooking, extrusion, and pelleting, may have detrimental effects on secondary protein components. These processes can cause protein denaturation, leading to the loss of native protein structure, alteration of functional properties, and reduced digestibility (Salazar-Villanea et al., 2016).

The study demonstrated that the digestibility of all fermented proteins increased. This finding indicates that fermentation positively impacts the breakdown and digestion of proteins, making them more easily digestible by the body. Furthermore, the study found that a decrease in the ratio of α-helix to β-sheet is associated with enhanced intestinal protein digestibility. The α-helix and β-sheet are secondary structures of proteins, and the ratio of these two components can influence the accessibility of digestive enzymes to the protein bonds during digestion. A decrease in the α-helix to β-sheet ratio suggests a structural change in the protein, potentially making it more susceptible to enzymatic hydrolysis and digestion (Alrosan et al., 2022; Salazar-Villanea et al., 2016).

The ratios of α-helix to β-sheet for LP-WP complexes decreased from 22.25 to 0.00% on the 2nd day of the fermentation. The reduction could be due to the fermentation having more effect on the complex protein than LPs. Microorganisms and their enzymes contributed to these results as they considerably influence secondary protein structure components (Yasar et al., 2020). These findings resemble the LP-WP complexes during fermentation (Table 2). Meanwhile, the α-helix percentage of LP-WP complexes has reduced more than the unfermented protein complex (Fig. 1A). This indicating the complexation of LPs with WPs played a significant role in the α-helix percentage during fermentation. LAB can degrade protein α-helix during fermentation by water kefir (Kieliszek et al., 2021; Savijoki et al., 2006).

### Effect of water kefir fermentation on phenolic compounds of fermented LP-WP complex

Phenolic compounds can bind to proteins through various mechanisms, including hydrogen bonding and hydrophobic interactions. This binding interaction can lead to the formation of protein–phenolic complexes, resulting in reduced protein solubility (Alrosan et al., 2023; Al‐Qaisi et al., 2024; Liu et al., 2023), altered protein structure, and impaired digestibility (Alrosan et al., 2022; Liu et al., 2023). The concentration of phenolic compounds in the LP-WP complex was assessed over 5 days during the fermentation process using water kefir (Table 3). The study’s findings indicate that the protein complexes’ TPC demonstrated a boost following the fermentation process. The increase in the TPC suggested that the water kefir fermentation process positively impacts the phenolic compounds in the LP-WP complex. The rise in TPC could be attributed to the breakdown of larger molecules during fermentation, leading to a higher concentration of phenolic compounds (Đorđević et al., 2010; Liyana-Pathirana and Shahidi., 2006). It was reported by Acosta-Estrada et al. (2014), Tu et al. (2019), Gunenc et al. (2017), and Alrosan et al. (2023) that fermentation by water kefir could increase the phenolic compounds during fermentation of fermented proteins. A study by Lai et al. (2013) showed that LAB increased the phenolic compounds in soy from 4.60 ± 0.28 to 5.96 ± 0.17 mg GAE/g during the fermentation.

**Table 3:** Changes in the total phenolic content (TPC, mg GAE/100 g) and phenolic compounds (mg/100 g) of unfermented LP-WP complexes (Day 0) and water kefir seed water kefir-fermented LP-WP complexes (Day 1 to 5)

	Fermentation period (Day)						*P*-value
	0	1	2	3	4	5	
TPC	223.73 ± 5.47^e^	265.52 ± 2.73^b^	279.85 ± 1.03^a^	236.27 ± 2.74^c^	232.09 ± 0.00c^d^	230.90 ± 1.03^d^	*P* < 0.05
Phenolic Compounds							
Catechin	17.97 ± 0.25^e^	33.44 ± 0.29^c^	34.48 ± 0.42^b^	38.27 ± 0.13^a^	33.15 ± 0.08^c^	32.21 ± 0.24^d^	*P* < 0.05
Chlorogenic	44.84 ± 0.52^e^	48.41 ± 0.41^b^	54.91 ± 0.22^a^	47.08 ± 0.15^c^	45.99 ± 0.78^c^	45.22 ± 1.16^de^	*P* < 0.05
Epicatechin	56.41 ± 0.29^c^	69.08 ± 0.31^b^	82.37 ± 0.45^a^	69.74 ± 1.59^b^	68.79 ± 0.94^b^	67.86 ± 1.78^b^	*P* < 0.05
Quercetin	7.42 ± 0.36^c^	7.65 ± 0.24^c^	8.24 ± 0.10^b^	9.36 ± 0.36^a^	9.19 ± 0.44^a^	1.89 ± 0.06^e^	*P* < 0.05
Rutin	1.28 ± 0.03^c^	1.03 ± 0.04^d^	1.04 ± 0.02^cd^	2.63 ± 0.14^a^	2.67 ± 0.04^a^	2.30 ± 0.30^b^	*P* < 0.05
Caffeic acid	ND	ND	ND	ND	2.52 ± 0.03^b^	3.30 ± 0.03^a^	*P* < 0.05
Ferulic acid	1.99 ± 0.02^d^	2.08 ± 0.04^c^	2.26 ± 0.02^b^	2.36 ± 0.02^a^	2.30 ± 0.03^b^	2.29 ± 0.02^b^	*P* < 0.05
Gallic acid	6.21 ± 0.47^f^	10.50 ± 0.23^c^	11.21 ± 0.21^b^	16.05 ± 0.37^a^	9.73 ± 0.52^d^	8.73 ± 0.27^e^	*P* < 0.05
Sinapic acid	ND	0.87 ± 0.02^ab^	0.76 ± 0.06^b^	0.67 ± 0.02^ab^	0.61 ± 0.02^ab^	0.56 ± 0.01^ab^	*P* < 0.05
Syringic acid	24.76 ± 0.51^b^	25.37 ± 0.27^b^	27.30 ± 0.32^a^	26.98 ± 0.32^a^	26.84 ± 0.95^a^	25.68 ± 0.62^b^	*P* < 0.05
Phenolic Compounds (Ʃ)	160.87^f^	198.42^d^	222.57^a^	213.13^b^	201.80^c^	190.04^e^	*P* < 0.05

The data presented in the table represent the mean ± standard deviation (n = 3). Values with different superscripts within the same row are statistically significant from each other (*P* < 0.05). ND represents not detected. LP-WP represents LP-WP complex at a ratio of 1:1.2

The syringic acid, gallic acid, epicatechin, catechin, caffeic acid, sinapic acid, ferulic acid, chlorogenic acid, quercetin, and rutin were increased during the fermentation by water kefir in the fermented protein complex (Table 3). Meanwhile, rutin in LP-WP complexes decreased from 1.28 ± 0.03 to 1.04 ± 0.02 mg/100 g on the 2nd day of the water kefir fermentation. The chemical composition of phenolics in plants determines their solubility, which can also range from simple to highly polymerized; chemical compounds could include phenolic acids, phenylpropanoids, anthocyanins, and tannins in different quantities (Garcia-Salas et al., 2010). Different phenolic compounds have varying chemical structures and properties, influencing their solubility and interactions with other molecules (Al‐Qaisi et al., 2024). Some phenolic compounds may be more readily extracted using certain solvents or extraction methods, while others may require different conditions for optimal extraction (Naczk & Shahidi, 2006).

The enzymes produced by microorganisms involved in fermentation, such as bacteria or yeast, possess specific capabilities to break down complex structures, including those formed by binding phenolic compounds with proteins or carbohydrates. These enzymes can cleave the chemical bonds between phenolic compounds and other molecules, releasing free phenolic compounds (Ajila et al., 2011; Santos et al., 2018). All phenolic compounds detected in these fermented protein complexes significantly (*P* < 0.05) differed during the fermentation.

The ability of LAB to metabolize tannins and phenolic acid esters through the action of tannases and related phenolic acid esterases is beneficial in the fermentation process of certain foods and beverages. LAB can modify taste, aroma, and texture by degrading these complex compounds, producing more palatable and well-balanced products. It was reported by Muñoz et al. (2017) that the presence and activity of tannases and related phenolic acid esterases can vary among different LAB strains. Some strains may exhibit higher enzymatic activity and efficiency in metabolizing tannins and phenolic compounds than others. Additionally, the fermentation conditions, such as pH, temperature, and substrate composition, can also influence the production and activity of these enzymes. LAB metabolize tannins by tannases and related phenolic acid esterases, which leads to an increase in the gallic acid from tannins, consequently decreasing the interaction between complex phenolic and protein and then the breakdown of easter (Fig. 1A). Phenolic compounds were found to be more abundant than the level of phenolic compounds identified in our study. This trend is because phenolic compounds are secondary metabolites in plants, and there are over 8000 different phenolic compound structures (Cosme et al., 2020). *Lactobacilli* is significant in generating phenolic acids by phenolic acid esterases that are esterified with plant cell wall polysaccharides (Muñoz et al., 2017).

In this study, the fermentation process can lead to the release and transformation of phenolic compounds, resulting in an overall increase in TPC. During water kefir fermentation, the microorganisms present in the kefir grains, including LAB and yeasts, can also contribute to releasing phenolic compounds from the substrate or the kefir grains themselves. This observation is consistent with the results that have been reported by Acosta-Estrada et al. (2014), Gunenc et al. (2017), and Tu et al. (2019). In addition, Lai et al. (2013) reported that LAB fermentation can increase soy’s phenolic content. The gallic acid content increased LP-WP complexes during fermentation could be due to tannins’ degradation by LAB’s tannases during the fermentation (Muñoz et al., 2017).

### Effect of water kefir fermentation on TSC of fermented LP-WP complex

Saponins are composed of a steroidal or triterpenoid aglycone (sapogenin) linked to one or more sugar moieties, such as glucose, galactose, or xylose, forming glycosidic bonds (Zhang et al., 2018). This study determined the TSC of LP-WP complexes throughout the 5 days of water kefir fermentation (Table 1). The TSC of LP-WP complexes was approximately 25.60 ± 0.57 OAE/100 g, which was significantly (*P* < 0.05) reduced to 22.10 ± 0.40 mg OAE/100 g on the 2nd day of water kefir fermentation and further reduced to 18.10 ± 0.72 mg OAE/100 g at the end of the fermentation. The saponins found in the LP-WP complexes are from LPs since lentils have high amounts of saponins (Del Hierro et al., 2018). A decrease in the TSC during water kefir fermentation could be attributed to the degradation of saponins connected to the protein and carbohydrate structures.

This outcome is consistent with other investigations demonstrating a decline in non-nutritive substances, such as tannins and saponins, following fermentation (Dajanta et al., 2011; Tu et al., 2019). These non-nutritive substances are often present in plant-based foods and can negatively affect digestion and nutrient absorption. Therefore, reducing these compounds through fermentation can potentially enhance the overall nutritional value of fermented protein. Our findings showed the reduction of TSC (Table 1) in LP-WP complexes on the 2nd day led to an increase in the digestibility of LP-WP complexes (Table 1). For example, the TSC decreased from 25.60 ± 0.57 to 22.10 ± 0.4 mg OAE/100 g of LP-WP complexes during the 2nd day of fermentation. There is no significant (*P* > 0.05) difference between the last 2 days (Day 4 and Day 5) of the water kefir fermentation. However, a significant (*P* < 0.05) difference exists when comparing these 2 days with the initial reading on Day 0. Our discoveries agreed with the protein digestibility of LP-WP complexes. Furthermore, the digestibility of LP-WP complexes increased from 81.69 to 88.84% after the reduction of TSC from 25.60 ± 0.57 to 22.1 ± 0.40 mg OAE/100 g on the 2nd day of fermentation. Several researchers have reported that reducing non-nutritive compounds increased protein digestibility (Hassan et al., 2015). In addition, it was also reported that a reduction in the saponins-crosslinked proteins could improve protein digestibility (Segal et al., 1970). Meanwhile, the TSC of LP-WP complexes has low saponins content because LP-WP complexes contain animal-based proteins.

### The dynamics of the growth fermenting microorganisms during water kefir fermentation

**Fig. 2:**
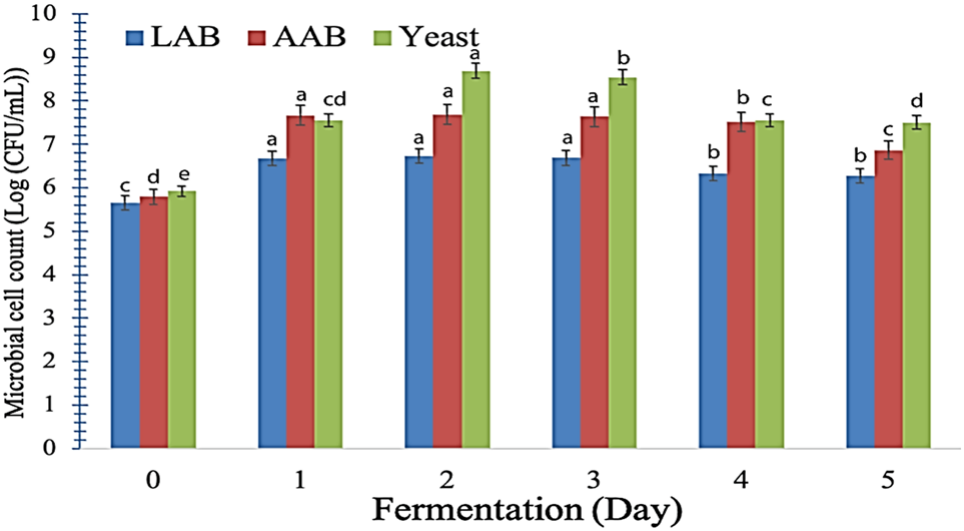
Illustration of the changes in the microbial activity of lactic acid bacteria (LAB), acetic acid bacteria (AAB), and yeasts during water kefir fermentation for 5 days at 25 °C. Values with different superscripts within the same color column are statistically significant from each other (P < 0.05). LP-WP represents LP-WP complex at a ratio of 1:1.2

This study examined the proliferation of AAB, LAB, and yeasts within the LP-WP complexes throughout the process of water kefir fermentation. The bacterial growth model illustrates the microbial interaction and fermentation pattern regarding substrate utilization and metabolomic growth (Fig. 2). This model is crucial for understanding the dynamics of microbial communities and their impact on complex proteins. By studying substrate utilization and metabolomic growth, scientists can develop strategies to optimize these processes and harness the potential of microorganisms for water kefir fermentation. Following 48 h of fermentation, the populations of yeasts and AAB showed a growth greater than 7 log CFU/mL. Conversely, the LAB achieved a population of 6.7 log CFU/mL within the same fermentation time frame.

LAB are a prominent group of microorganisms found in water kefir grains. They include *Streptococcus, Leuconostoc, Lactococcus,* and *Lactobacillus*. LAB play a crucial role in water kefir fermentation by converting sugars into lactic acid, contributing to fermented protein’s sourness and acidity. These bacteria also produce other metabolites, such as acetic acid, ethanol, and various flavor compounds, adding complexity to the sensory profile of water kefir (Tu et al., 2019). In this study, the LAB achieved a population of 6.7 log CFU/mL on the 2nd day of the water kefir fermentation. AAB are another group of fermenting microorganisms present in water kefir grains. They convert ethanol into acetic acid, contributing to the fermenting medium’s tangy flavor and acidity. AAB commonly found in water kefir include species like *Acetobacter* and *Gluconobacter* (Tu et al., 2019; Alrosan et al., 2023). Following 48 h of fermentation, the populations of AAB showed a growth greater than 7 log CFU/mL.

Yeasts are essential for the fermentation of water kefir as they metabolize sugars, producing carbon dioxide and alcohol. Various yeast species exist in water kefir grains, including *Saccharomyces*, *Candida*, and *Kluyveromyces*. These yeasts contribute to the carbonation and alcohol content of the beverage, along with producing flavor and aroma compounds that enhance the sensory characteristics of water kefir (Tu et al., 2019; Alrosan et al., 2023). The yeast populations showed a growth greater than 7 log CFU/mL. Several studies reported that the composition of microorganisms in water kefir grains can vary depending on factors such as the source of the grains and the fermentation conditions (Gulitz et al., 2011; Randazzo et al., 2016; Alrosan et al., 2022). This variability can lead to differences in the microbial profile and the sensory attributes of the resulting water kefir. Overall, the presence of LAB, AAB, and yeasts in this study contributes to the diverse microbial ecosystem of water kefir grains and plays a vital role in the fermentation process and the characteristics of the fermented LP-WP complex.

In conclusion, water kefir fermentation was successfully conducted on LP-based protein complexes, specifically LP-WP-1:1.2. Water kefir fermentation refers to the process of utilizing water kefir grains to ferment a liquid medium containing protein complex made of LPs and WPs. LP-WP-1:1.2 refers to a specific ratio of LPs to WPs complexes used as the substrate for water kefir fermentation. During water kefir fermentation, the microorganisms in the water kefir metabolize the available nutrients, including the proteins in the LP-WP complexes. The metabolic activities of the fermenting microorganisms, such as LAB, AAB, and yeasts, contribute to the breakdown and transformation of the LP-WP complexes. Through enzymatic reactions and metabolic processes, the microorganisms release enzymes that can hydrolyze protein complexes, facilitating the hydrolysis of proteins into smaller peptides and amino acids. These smaller protein fragments are more readily digestible and can be further utilized by the microorganisms for their growth and metabolism. These changes could modify the LP-WP complex’s composition, structure, and nutritional properties, potentially impacting digestibility, functionality, and bioactivity.

## Data Availability

Available on request.
